# Clinical Applications of Synovial Biopsy

**DOI:** 10.3389/fmed.2019.00102

**Published:** 2019-05-10

**Authors:** Antonio Manzo, Serena Bugatti, Silvia Rossi

**Affiliations:** Rheumatology and Translational Immunology Research Laboratories, Division of Rheumatology, IRCCS Policlinico San Matteo Foundation, University of Pavia, Pavia, Italy

**Keywords:** synovial biopsy, arthritis, synovitis, biomarkers, precision medicine

## Abstract

The synovial tissue is a primary target of multiple diseases characterized by different pathogenic mechanisms, including infective, deposition, neoplastic, and chronic immune-inflammatory pathologies. Synovial biopsy can have a relevant role in differential diagnosis of specific conditions in clinical practice, although its exploitation remains relatively limited. In particular, no validated synovial-tissue-derived biomarkers are currently available in the clinic to aid in the diagnosis and management in most frequent forms of chronic inflammatory arthropathies, namely rheumatoid arthritis (RA) and the spondyloarthritides (SpA). In this brief review, we will discuss the current spectrum of clinical applications of synovial biopsy in routine rheumatologic care and will provide an analysis of the perspectives for its potential exploitation in patients with chronic inflammatory arthritides.

The assessment of the pathologic process at peripheral sites has been proved as a source of clinically relevant information in different human pathologies including cancers and systemic autoimmune diseases. Examples of the latter group are the assessment of the salivary glands in sialo adenitis, the muscle in idiopathic inflammatory myopathies or the kidney in systemic lupus erythematosus, in which the qualitative and/or quantitative analysis of local inflammatory processes can be exploited to corroborate diagnosis/classification, facilitate discrimination among disease entities, evaluate prognosis and guide the choice of appropriate treatments (1–3). Similarly, the synovial membrane, being the target of different rheumatologic conditions, holds an intrinsic potential for wide clinical applications, although its exploitation remains, at present, relatively limited. If, on the one hand, the synovial biopsy may offer unique information aiding the diagnosis of infectious and other rare diseases, on the other, no validated synovial tissue-derived biomarkers are currently available in the clinic to support early diagnosis/classification or to guide individual patients' management in most frequent forms of chronic inflammatory arthropathies.

Based on the existence of major unmet needs and on data derived from previous proof-of-concept studies (see next paragraphs), there is now growing attention in expanding the translational applicability of synovial tissue analysis also in this direction (4). One of the most compelling working hypothesis is that the cellular/molecular patho-biology of the inflamed synovial membrane might delineate specific discriminative traits able to improve early diagnosis of undifferentiated forms and patients' stratification into treatment-specific response groups. The introduction of mini-invasive approaches allowing targeted tissue sampling of large and small joints under direct vision of a standard ultrasound (US) machine (US-guided biopsies) is now contributing to make this perspective more realistic, favoring synovial biopsy widespread applicability and allowing the development of multi-center research and clinical trials (5–10). For a detailed update on currently available synovial biopsy techniques, including their advantages, limitations and validation requirements the reader can refer to a recently published review (11).

In the following paragraphs, we will provide a summary of current applications of synovial biopsy in clinical practice and of the background data that are allowing to conceive their extension into the field of stratified medicine.

## Current Clinical Applications of Synovial Biopsy: Differential Diagnosis in Routine Care

For the majority of rheumatologic diseases, patients' interview, clinical examination, imaging and serological tests are usually sufficient to establish a diagnosis and monitor treatment response. The analysis of the synovial tissue can be, however, of assistance for diagnostic purposes in course of arthritis of undetermined origin, allowing the identification of specific traits of a restricted, though defined, spectrum of pathologies, including infective, neoplastic and some deposition diseases (Table 1). Whilst specific markers (cellular and/or molecular) related to several of these conditions can be readily identified also through less-invasive approaches, such as the analysis of synovial fluid, the biopsy can be a relevant implementation tool in different situations. Firstly, the collection of synovial tissue can be essential to ensure sampling of joint environments characterized by lack of or limited effusion, as a primary approach or as a “failsafe” mechanism (12, 13). Under certain circumstances, the synovial biopsy can be important also as a complementary or second level approach in the case of fluid availability. Indeed, despite comparative data on fluid vs. tissue diagnostic accuracy for most conventional approaches (microbiological cultures, PCR for infective agents, detection of crystals) remain limited (14–17), results derived from the two compartments, even if focused on the same downstream procedure, have been shown to lack systematic redundancy and either may contemplate false negative results (13–21).

**Table 1:** Clinical utility of synovial biopsy in differential diagnosis.

Deposition diseases	Crystal arthropathies Amyloidosis Ochronosis Hemochromatosis
Infectious arthritis	Low-grade infections by common bacteria Mycobacterial arthritis Spirochetal arthritis (Lyme disease, syphilis) Whipple's disease Fungal arthritis
Synovial tumors	Synovial cell sarcoma/synovial chondrosarcoma Lymphoma and metastatic carcinoma Pigmented villonodular synovitis Synovial chondromatosis
Histiocytic disorders and others	Multicentric reticulohistiocytosis Erdheim-Chester disease Chronic sarcoidosis Foreign-body arthritis

Beyond expanding the analytical substrate, the availability of biopsy specimens may also offer specific information by allowing the integration of microbiological and molecular screenings with the analysis of characteristic histopathologic traits of some infections and rare diseases (see next paragraphs for details).

### Deposition Diseases

US-guided dry needle synovial tissue aspiration (22) or synovial biopsy (23) can be considered as diagnostic options when crystal-associated arthropathies are suspected, in particular in patients without synovial effusion or in the case of negative results from synovial fluid. Both monosodium urate (after tissue fixation in absolute alcohol) and calcium pyrophosphate crystals (24) can be detected within tissue specimens as focal deposits of amorphous material or as birefringent structures by polarized light microscopy. As a general notion, inferred from a recent retrospective study of biopsy reports involving synovial tissue between 1998 and 2015, a confirmatory diagnosis of crystal associated arthritis can be established in ~40% of the cases in which the procedure is performed for a primary clinical suspicion (23).

Both synovial fluid or synovial tissue analyses can also contribute to the differential diagnosis of other rarer deposition diseases including amyloid arthropathy, through Congo-red stain and the identification of typical apple-green birefringent deposits of immunoglobulin free light chains, as well as ochronotic arthritis, typically associated to local accumulation of homogentisic acid polymers and characteristic yellow-brown cartilage debris (25–28).

### Infectious Arthritis

In keeping with deposition diseases, the diagnosis of suspected infectious arthropathies can be approached either through the analysis of synovial fluid or synovial tissue. Both type of samples have been successfully exploited for the identification of pathogenic organisms through microbiological cultures and molecular analyses. Unfortunately, due to the scarcity of systematic studies, no definite guidelines are currently available to assist clinicians in the selection of the most appropriate strategy when both sites are accessible. Notwithstanding this gap, the availability of synovial specimens can represent, however, a benefit in certain circumstances by offering a wider spectrum of analytical perspectives. These perspectives, which may be of particular relevance in the case of negative results from cultural examinations, include the direct analysis of bacterial and fungal localization *in situ* through conventional stainings (Gram, Ziehl, Dieterle, periodic acid-Schiff), as well as the evaluation of indirect signs, such as the presence/pathologic aspect of local granulomatous reactions or the degree of perivascular neutrophilic infiltration (29, 30). The latter parameter, though not specific *per se*, has been reproducibly shown to be a valid discriminative marker of septic arthritis if quantitatively addressed, either through conventional haematoxylin and eosin (H&E) stain or CD15 immunohistochemistry (31–34).

Broad-range 16S rRNA bacterial PCR has not proved to offer major advantages over bacterial culture in the standard diagnostic setting (35) and is considered prone to non-specific results (36). It has been however proposed as a candidate method to monitor the presence of bacterial DNA in synovial samples from patients with septic arthritis during antibiotic treatment (19). Targeted-PCR testing for mycobacteria and difficult-to-culture atypical germs (Borrelia, Tropheryma whipplei) has been similarly applied to both synovial fluid and synovial membrane and can be considered in the case of suspicion of mycobacterial- (37, 38), Lyme- (20, 39) and Whipple's arthritis (20, 40) when a sensitive approach is required.

### Synovial Tumors and Histiocytic Disorders

Tissue-directed analyses give also the unique opportunity to broaden the diagnostic spectrum in patients with unclassified arthritis, allowing the identification of specific (non-infective) conditions characterized by typical synovial histopathologic features and less traceable changes in the synovial fluid. Examples of these conditions, in which the synovial biopsy may have a primary diagnostic role, include primary synovial malignancies (lymphomas, sarcomas), metastatic tumors and some benign proliferative lesions like pigmented villonodular synovitis and synovial chondromatosis (the latter characterized by a minor risk of malignant transformation). In all these conditions, the *in situ* evaluation by conventional histopathologic analyses can be required to integrate and corroborate imaging findings for a defined differential diagnosis (20, 41–43).

The standard histologic analysis of the synovium can be instrumental also in the diagnosis of arthritis in patients affected by some non-Langerhans cell histiocytic disorders, uncommon conditions characterized by multi-system involvement due to dysregulated accumulation of mononuclear phagocytes. In some forms with adult-onset (multicentric reticulohistiocytosis, Erdheim-Chester disease), patients can display severe joint involvement, typically associated to abnormal sub-lining infiltration of CD68-positive (CD1a- and S100-negative) histiocytes and multinucleated giant cells with a lipid-laden or PAS-positive ground-glass cytoplasm (44–46).

### Diagnostic Value of Synovial Biopsy in Real-Life Clinical Practice

Altogether, these data, generated over the last decades, demonstrated the utility of synovial tissue collection for differential diagnosis, but left partially unclear the actual output of the procedure in the real-life setting of a rheumatology clinic, in particular for what concerns most recent approaches, such as US-guided biopsy. This issue has now been addressed by independent groups demonstrating, quite consistently, a success rate of around 82–96% in obtaining samples suitable for analysis with the potential to achieve a diagnosis in between 16 and 20% of the cases (13, 20, 47), depending on the inclusion criteria and study design (13, 20, 47).

## Translational Applicability of Synovial Biopsy in Clinical Trials: Surrogate Biomarkers of Clinical Response to Treatment

Beyond its possible application for differential diagnosis of unclassified arthritis, synovial tissue examination has been also proved to be a valuable source of surrogate biomarkers of response-to-treatment. Evidence supporting this concept derives from pioneering studies performed in the last two decades demonstrating, through the evaluation of serial arthroscopic biopsies, the sensitivity-to-change and external responsiveness of sub-lining CD68+ macrophages in relationship to variations of clinical composite indices in rheumatoid arthritis (RA). The reduction of CD68+ sub-lining macrophages has been shown to be associated to effective treatment, less influenced by placebo compared to clinical parameters, and to be a valid measure of response to treatments characterized by different mechanisms of action (48–51). Collectively, these observations have corroborated the value of synovial tissue analysis for the development of markers of early patho-biologic effect, thus potentially exploitable to accelerate decisions (including dose selection) in early phase I/II clinical trials. Strengthening the general applicability of these data, the correlation between modulation of the number of CD68+ sublining macrophages with clinical response to treatment has been recently confirmed also through the assessment of US-guided biopsies restricted to tissue collection from small joints (8).

## Clinical Perspectives: Early Diagnosis of Chronic Inflammatory Arthritides

If, on the one hand, the studies presented in the previous sections delineated the conditions that can be diagnosed through a synovial biopsy, on the other, they also shed further emphasis on what synovial tissue sampling cannot currently offer in routine clinical practice. The possibility to identify specific traits for most common forms of systemic chronic inflammatory arthropathies, namely RA and spondyloarthritis (SpA) remains, indeed, impracticable. This issue is relevant both in patho-biologic and clinical terms and has been the object of intense investigation in the past. Indeed, since early diagnosis and treatment in these conditions are linked to improved long-term outcomes (52, 53), the identification of disease-specific pathologic changes would contribute not only to improve comprehension of disease pathogenesis but, potentially, also to improve current models for early outcome prediction in undifferentiated forms (54–56).

RA and SpA synovitis (evaluated at a group level) do display measurable differences compared with post-traumatic and degenerative conditions, in terms of gene expression (57), histopathologic score (Krenn's, IMSYC) (58, 59), and cell proliferation rate (60). None of the analyzed parameters, however, has so far proved a sufficient degree of diagnostic accuracy due to intra-disease variability and overlapping features. The same concept applies for what concerns the overall level of micro-anatomic organization of inflammatory infiltrate that has been shown, as expected from studies in different pathologic contexts (61), to present similar qualitative characteristics (62, 63).

Despite these data and the observed gross analogies, there is now growing evidence from independent studies that a detailed comparative analysis of specific components of the inflammatory process may actually allow to detect multiple and congruent biological differences among diseases, in particular if patients' characteristics and the overall degree of joint inflammation are appropriately matched. One of the most compelling aspects that has been reproducibly confirmed relates to the characteristics of the vascular system. Synovial vascularity has been shown to display macroscopic and microscopic differences between RA and SpA, with the latter associated to an increased distribution of tortuous blood vessels in the sub-lining both in early and established disease (64–67). Accordingly, the level of synovial production of angiogenic factors (VEGF and Ang2 mRNA and protein) is significantly increased in psoriatic arthritis (PsA) compared to RA, with a prominent differential expression in perivascular regions. Since Ang2 expression in the presence of VEGF is functionally implicated in angiogenesis and vessel destabilization, it has been proposed that the observed high levels of Ang2/VEGF in PsA joint could inhibit stabilization of the new vessels, resulting in the formation of more “plastic” vessels (68).

The existence of peculiar biological traits characteristic of SpA synovial stroma has been confirmed by gene expression analyses. In this context, of particular interest is the work performed by Yeremenko et al. (69) who, by pan-genomic microarrays of synovial samples from patients with SpA and RA matched for the local degree of histological inflammation, demonstrated a robust disease-specific, inflammation-independent myogene expression signature in SpA synovitis. Synovial tissue staining identified the myogene expressing cells as α-SMA positive, vimentin-positive, prolyl 4-hydroxylase-positive, CD90+ and CD146+ mesenchymal cells, confirming their significant over-representation in the lining and sub-lining of the inflamed SpA synovium.

No differential characteristics, instead, have been reproducibly recognized in the distribution of major lymphocyte populations (conventional CD3+ T cells, CD8+ T cells, B cells, plasma cells) and of lining/sub-lining CD68+ macrophages (65–67, 70), although an increased prevalence of alternatively activated CD163+ macrophages (67, 71) and IL17 producing mast cells (72) has been reported in SpA.

In conclusion, data derived from several independent studies demonstrate that, despite a shared inflammatory background, the inflamed synovium of different forms of chronic inflammatory arthritides can associate to differential cellular and molecular traits. Further research and novel multi-center observational studies (56, 73) are needed to improve our mechanistic comprehension of these traits and delineate their predictive value in real-life clinical practice.

## Clinical Perspectives: Patients' Stratification Within and Across Chronic Inflammatory Arthritides

If differences in the synovial characteristics can be captured between different clinical entities, a cutting-edge question is whether clinically relevant differences can be reliably distinguished also within the same disease, a fundamental premise to conceive the possible integration of synovial biopsy into a precision medicine algorithm.

Precision medicine is an approach to disease treatment and prevention that takes into account individual patho-biologic variability, thus allowing to predict more accurately which treatment or prevention strategy for a particular disease will be more suitable in specific groups of patients. This perspective, which differs substantially from conventional approaches based on the “average person,” represents a major objective of modern healthcare systems due to both clinical and socio-economic needs. Chronic inflammatory arthritides have several characteristics that make them ideally suitable for stratification. These include the high degree of clinical heterogeneity that characterizes both RA and SpA, the degree of variability of response to specific treatments within each disease, and the similar degree of efficacy of specific treatments across individuals affected by different diseases (74). Whilst a rudimental level of stratification is already applied to RA, through the distinction of autoantibody-positive and -negative sub-groups (75), it is quite clear that these categories, *per se*, are not sufficient to entirely explain the heterogeneity of the disease and that a finer profiling is required (76). Since the synovial membrane represents one of the primary targets of these conditions, it is expected that dissecting its pathologic traits could be a privileged window on disease pathogenic spectrum (4).

Several studies performed in recent years have set the technical, pathological and clinical bases to support the scientific rationale of exploiting synovial biopsy for a precision medicine approach to arthritis.

In technical terms, the collection of a limited amount of tissue from a single procedure has been proved sufficient to obtain a reliable assessment of different histopathologic markers (6, 8, 77–79) and gene expression (80) in one joint. Despite differences among studies, depending on the adopted technique and measurement unit (number of specimens, mm^2^), all reported data were falling within the feasibility range of a routinely applicable procedure. As a complementary observation, the assessment of different characteristics (selected histopathologic markers and of T cell clonal expansions) in one joint has been shown to be representative of the same parameters in other joints in RA (81, 82). Notwithstanding the possible existence of variability in transcriptome signatures and epigenetic traits among different sites (83), current results collectively suggest that the analysis of few synovial specimens from a single accessible site can be informative on the systemic process.

In pathological terms, there is now extensive evidence indicating that the cross-sectional evaluation of the synovium from a single joint does actually allow the identification of defined inter-individual differences in RA. This concept has been supported by independent studies focused on different analytical perspectives: immuno-histology (84, 85), gene expression profiling of whole tissue (86–92), and RNA-seq data from isolated synovial cells (93).

A critical issue remains the interpretation of the observed heterogeneity and two main models are currently emerging. In particular, whilst some studies have described the variability of synovial characteristics primarily as a function of the overall degree of inflammation intensity (92, 94), other analyses have proposed the existence of a more qualitative spectrum, with the identification of distinct synovitis categories, each characterized by congruent histological, molecular and cytological correlates (95, 96). Based on the relative enrichment of specific gene sets, these categories have been defined by Dennis et al. (95) as: (i) *the lymphoid phenotype*, enriched in genes related to B-T lymphocyte activation-differentiation, immunoglobulin production and antigen presentation; (ii) *the myeloid phenotype*, also characterized by processes associated with TNFα and IL-1β production, TLR and NOD-like receptor signaling, Fcγ-receptor-meditated phagocytosis; (iii) *the fibroid phenotype*, enriched for genes associated with TGFβ and BMP signaling, together with SMAD binding, but lacking enrichment of any immune system processes; (iv) *the low inflammatory phenotype*, showing only enrichment for inflammatory and wound response processes. These phenotypes, or similar patterns according to a recently revised classification (97), have been shown to present measurable associations with specific biomarkers in peripheral blood (CXCL13 and soluble ICAM-1 for the lymphoid and myeloid phenotype, respectively) and to be detectable in early-untreated RA, strengthening their differential biologic impact also at systemic level and in the absence of treatment biases (95, 97).

In clinical terms, despite it remains unclear whether the heterogeneity of synovial features does reflect fixed characteristics of specific disease subsets or dynamic phases conditioned by fluctuations of the inflammatory process, we have now proof-of-concept evidence that the assessment of synovial inter-individual differences does actually have the potential to predict clinically-relevant outcomes. Data supporting this idea derive from independent observational studies based on patients' stratification through either histological parameters or molecular signatures. Associations between synovial pathologic traits and clinical response to specific treatments has been obtained in studies focusing on agent targeting different molecular pathways, including anti-TNF (95, 98–104), IL-6 inhibitors (105), or B cell depleting agents (106–108), pointing at a wide spectrum of applicability. The assessment of synovial patho-biology in single joints has been shown also to hold an intrinsic potential for the development of prognostic biomarkers, as it can be inferred, for example, by the association between B cell-rich/lymphoid synovitis (109) and radiographic progression, recently confirmed in independent RA cohorts (85, 97, 104).

### Is a Multidimensional Approach Required for Stratification of Systemic Inflammatory Arthritides?

**Figure 1:**
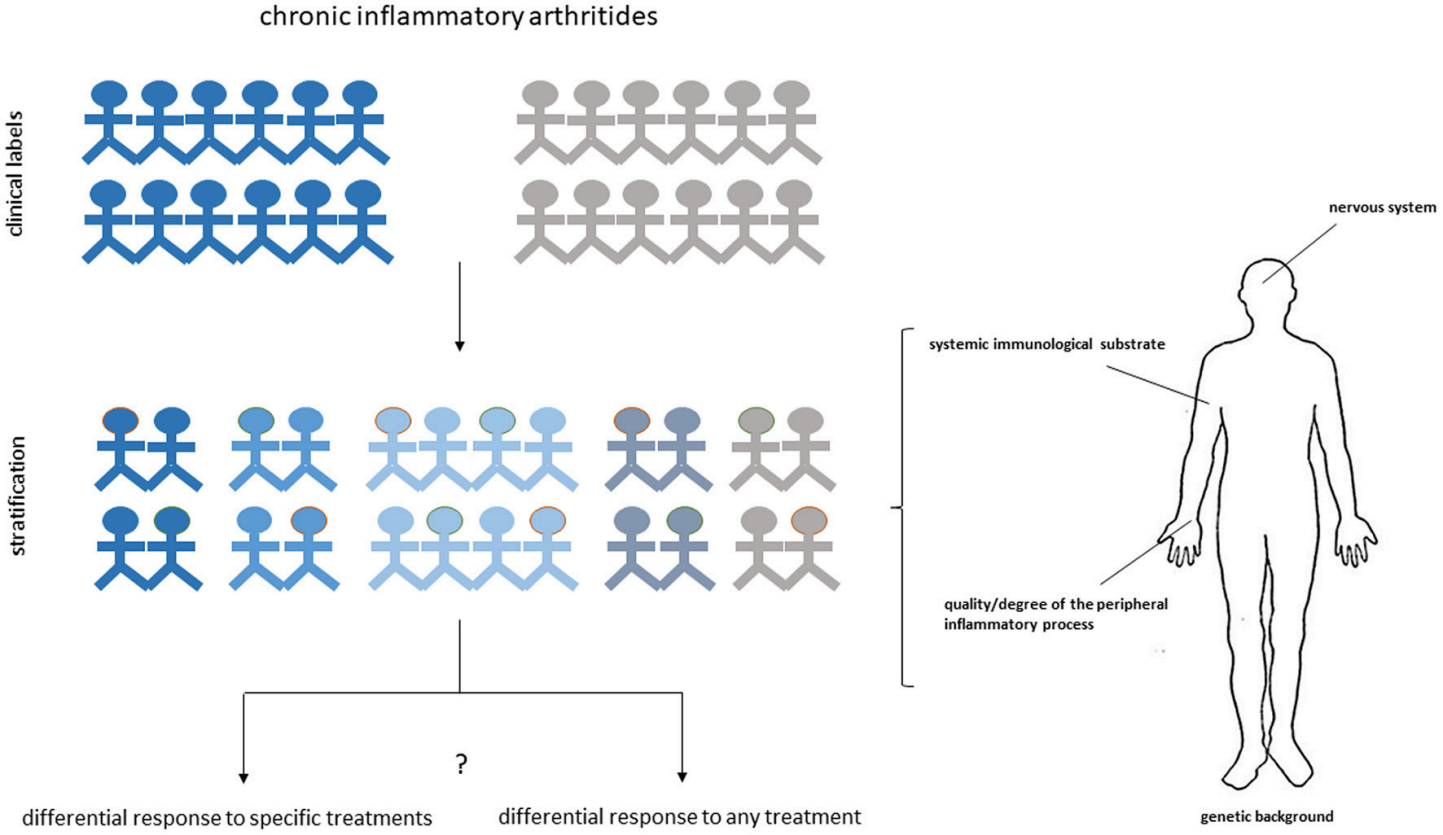
Multidimensional approach for personalized medicine in arthritis. Different anatomic and functional dimensions can cooperate to delineate the heterogeneous phenotype of chronic inflammatory arthritides and the predisposition of subgroup of patients to respond to specific treatments or to respond to any treatment. Dissecting the synovial histological and molecular characteristics of each individual patient may provide a fundamental contribution to the stratification process by offering a privileged window on the differential expression of the disease at target sites.

Despite current advancements, the development of a valid personalized approach to RA or SpA, based on synovial biopsy and applicable at community level still remains a very ambitious target. It should be indeed emphasized that data derived from available prediction studies, though promising, did not always led to univocal conclusions. Although differences might be obviously related to the limited sample size, differences in the definition of exposure variables and pre-set confounders, we might also consider that both RA and SpA are likely to be determined, as the majority of immune-inflammatory diseases, by a complex series of events controlled by polygenic, environmental and endocrine factors (110, 111). Some of these events might express themselves also at systemic level and in different anatomic compartments (112–115), providing a source of variability that may be missed by restricting the analysis to downstream inflammatory reactions. Response to treatment can be also influenced by patient-related subjective factors not directly reconcilable to measurable peripheral events (116). Thus, unlike oncology, in which the conception of a precision approach can be primarily based on genetic drivers, the approach to systemic immune-inflammatory diseases might require considering additional levels of complexity through the integration of different systems and clinical parameters (117) (Figure 1). A direct example supporting this hypothesis derives from the work performed by Lauwerys et al. demonstrating that the diagnostic accuracy of synovial analyses based on gene expression data increases from 56.8 to 98.6% by the addition of specific clinical symptoms in the prediction algorithm (57). Large size prospective multi-center clinical trials testing the relevance of biopsy-based patient stratification are currently in progress and are expected to offer direct insights into the actual predictive weight of synovial biopsy.

## Conclusions

Taken together, the studies discussed in this review highlight the important, though circumstantial role that synovial biopsy can have in current clinical practice. Depending on the clinical context, it may complement and in some cases substitute less invasive procedures, offering the possibility to integrate microbiologic and histopathologic data. The combination of these approaches in certain circumstances can be essential to achieve a definite diagnosis in patients with arthritis of undetermined origin. The spectrum of applicability of synovial biopsy remains, however, relatively limited mostly due to the lack of validated markers for the diagnosis and management of major forms of chronic inflammatory arthritis.

The next challenge is thus to define the exploitability of the heterogeneous molecular and cellular patterns that characterize the synovial tissue in RA and SpA for the development of novel diagnostic markers and multi-dimensional precision medicine algorithms. Based on recent data from observational studies and the technological advancements in synovial tissue sampling and analysis this perspective seems now more realistic. Of considerable relevance in this direction is the recent introduction of novel cutting-edge tools allowing transcriptional profiling and single-cell RNA sequencing of infiltrating cells isolated from synovial samples (118, 119). This technology, which has already contributed to the achievement of important goals in the characterization of novel cell subsets in RA (93, 120, 121), is expected in the near future to play a key role also in the field of biomarker discovery and in clinical translation. It is indeed likely that, compared to whole-tissue gene expression analyses, the assessment of synovial characteristics at single cell level might dramatically expand our possibilities to screen specific aspects of the pathogenic process and to unravel intrinsic characteristics of the disease. The fine deconstruction of the histopathological, molecular and cellular heterogeneity of the synovial inflammatory process by means of integrated high-throughput approaches might also lead in the near future to a novel taxonomic classification of chronic inflammatory arthritides, firmly rooted in basic pathogenic processes and, possibly, spanning across the boundaries of conventional clinical labels.

## Author Contributions

AM, SB, and SR contributed to literature review and preparation of the manuscript.

### Conflict of Interest Statement

The authors declare that the research was conducted in the absence of any commercial or financial relationships that could be construed as a potential conflict of interest. The reviewer AN declared a past co-authorship with one of the authors AM to the handling editor.
